# Retro-Odontoid Pseudotumor in a Patient with Atlanto-Occipital Assimilation

**DOI:** 10.5334/jbsr.1587

**Published:** 2018-10-02

**Authors:** Arvy Buttiens, Jan Vandevenne, Sofie Van Cauter

**Affiliations:** 1 Ziekenhuis Oost-Limburg, BE; 2 University Hasselt, BE

**Keywords:** Odontoid process, transverse ligament, atlanto-axial subluxation, rheumatoid arthritis, pseudotumor

## Abstract

A retro-odontoid pseudotumor is an uncommon non-neoplastic mass. They are mostly associated with rheumatoid arthritis and atlanto-axial subluxation. The pathogenesis is degeneration of the transverse ligament due to chronic mechanical stress. In this case report, an atlanto-occipital assimilation altered the biomechanics of the cervical spine, causing chronic mechanical stress on the transverse ligament and subsequently the development of a retro-odontoid pseudotumor. This is in accordance with previous studies that have attributed the development of retro-odontoid pseudotumor to a loss of mobility of the cervical spine, in cases without associated rheumatoid arthritis or atlanto-axial subluxation.

## Introduction

A retro-odontoid pseudotumor is a non-neoplastic mass posterior to the odontoid process of the axis. Compression of the myelum with myelopathy and severe neurological symptoms is a serious concern due to the location of the mass.

## Case Report

**Figure 1:**
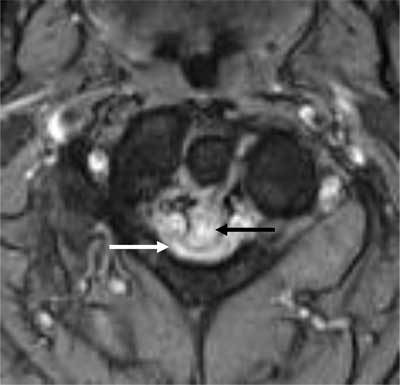
Axial T2-weighted image. The retro-odontoid pseudotumor is seen as a hyperintense mass (black arrow) which extrudes through the transverse ligament and compresses the myelum (white arrow).

**Figure 2:**
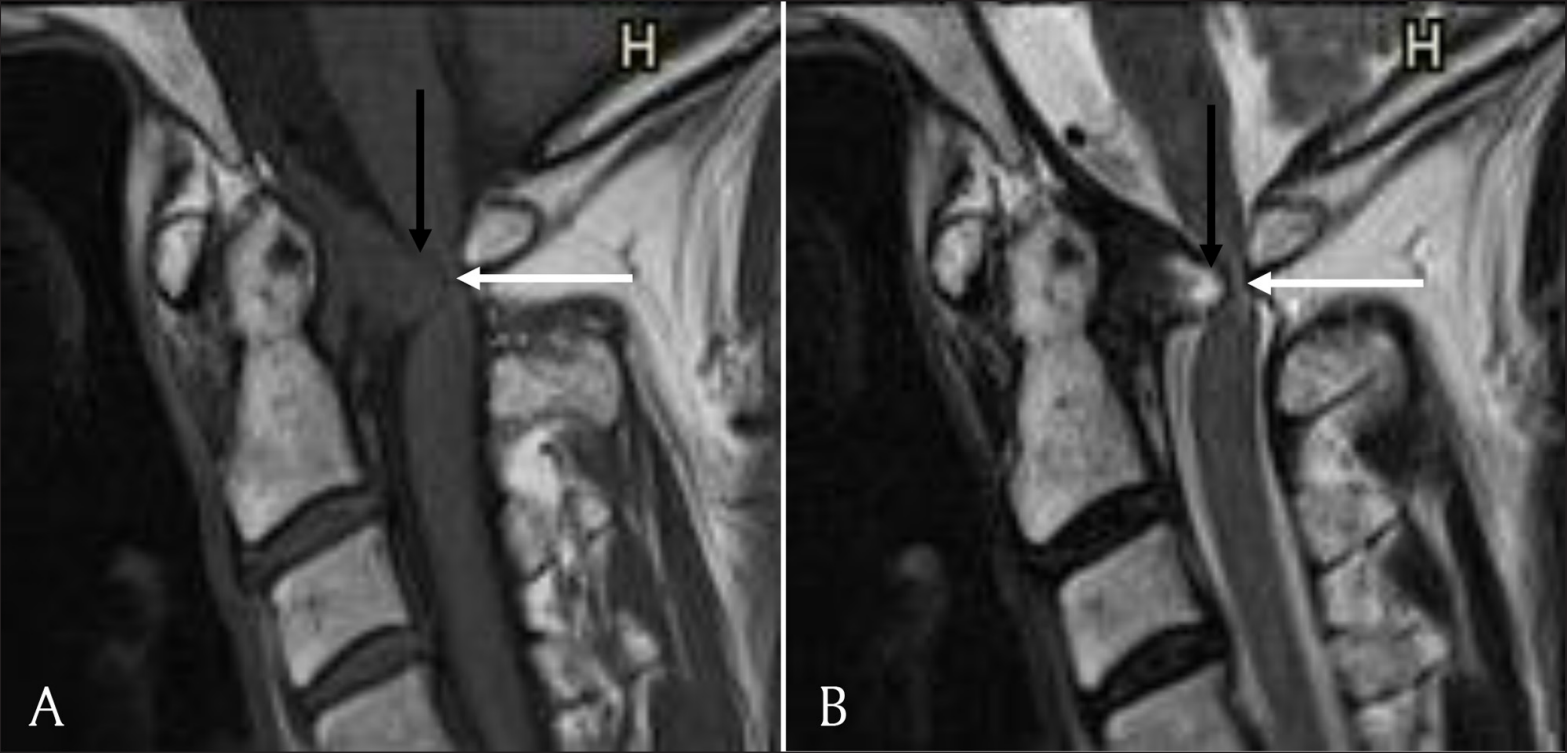
**(A)** Sagittal T1-weighted image. The retro-odontoid pseudotumor is seen as a mass (black arrow) extruding through the transverse ligament and compressing the myelum (white arrow). It has an isointense signal compared to the myelum. **(B)** Sagittal T2-weighted image. The retro-odontoid pseudotumor has a hyperintense signal (black arrow) and compresses the myelum (white arrow).

A 67-year-old female, with a typical presentation of carpal tunnel syndrome in the right hand, presented initially with numbness in digit IV and V a year later. A few months later, she developed numbness in all five fingers on the left side. Furthermore, the patient mentioned that she had had a burning sensation in both feet for a few years. Another few months later she complained of weakness in all four limbs and was sent for a neurological consultation with electromyography. Clinical examination revealed a Hoffmann-Trömner reflex on the left side, mild loss of strength in both hands, loss of vibratory sense and hypoesthesia in the distal end of all four limbs and loss of proprioception in both legs. The electromyography showed disturbed sensorimotor signals in the left hand and mildly disturbed sensorimotor signals in the right hand. The disturbed electromyography was attributed to carpal tunnel syndrome, which was presumably less severe on the right side due to treatment with long acting corticosteroid injections. The paresthesia in digit IV, digit V and both feet, however, could not be explained with the diagnosis of carpal tunnel syndrome. Therefore, magnetic resonance imaging (MRI) of the cervical spine was performed. Imaging showed an extra-dural soft tissue mass posterior to the odontoid process of the axis. The mass extruded through the transverse ligament of the atlas with severe compression of the myelum and myelomalacia at the level of C1 (Figures 1, 2A and 2B).

**Figure 3:**
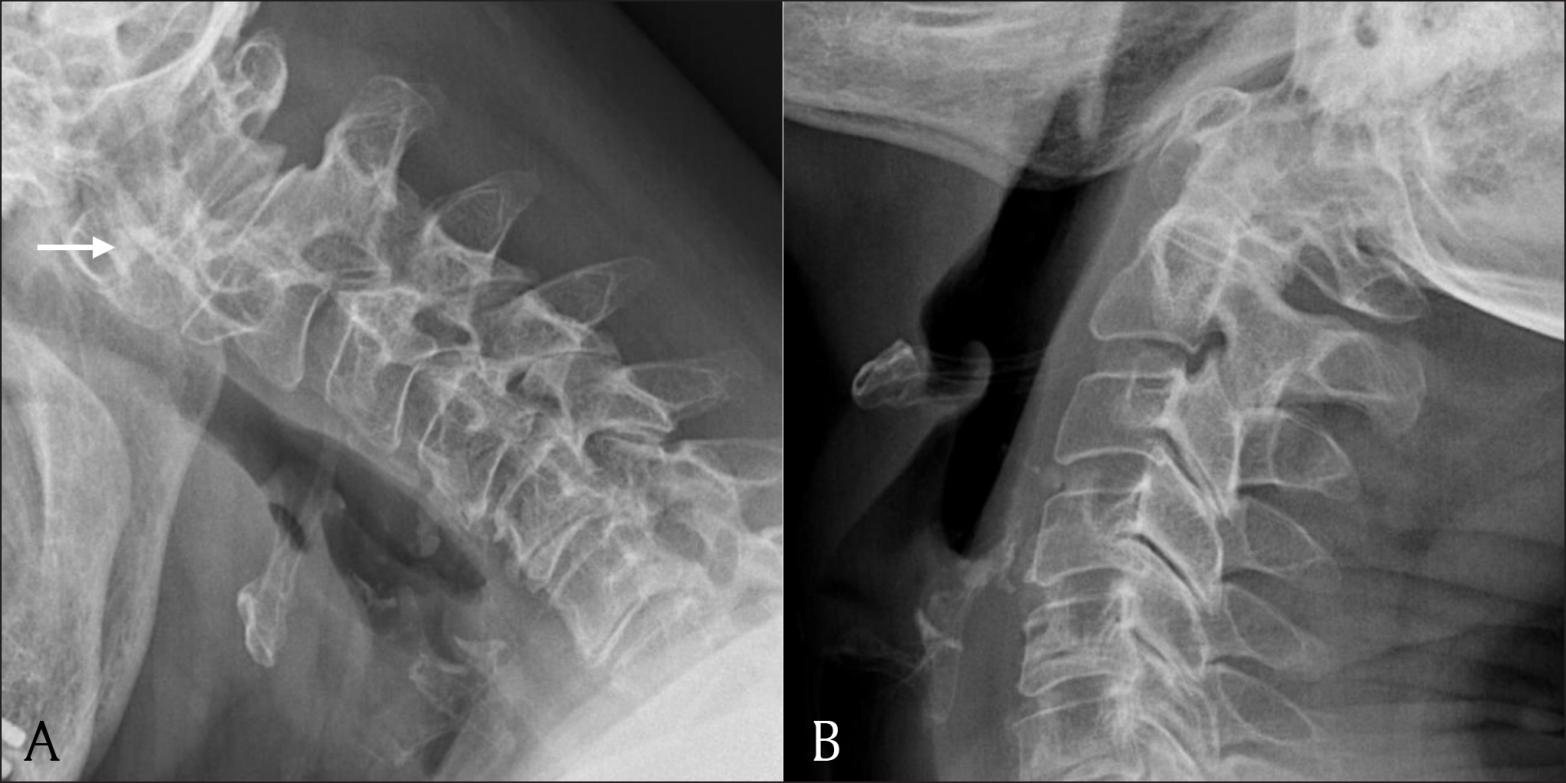
**A** = lateral view of the cervical spine in flexion; **B** = lateral view of the cervical spine in extension. Plain radiograph showing a lateral view of the cervical spine in flexion and extension. Note widening of the atlantodental interval in flexion (white arrow).

A plain radiograph of the cervical spine was performed to check for atlanto-axial instability. The radiograph during flexion shows a slightly widened atlantodental interval, measuring 4 mm (normal value: <3 mm) (Figure 3A and B).

**Figure 4:**
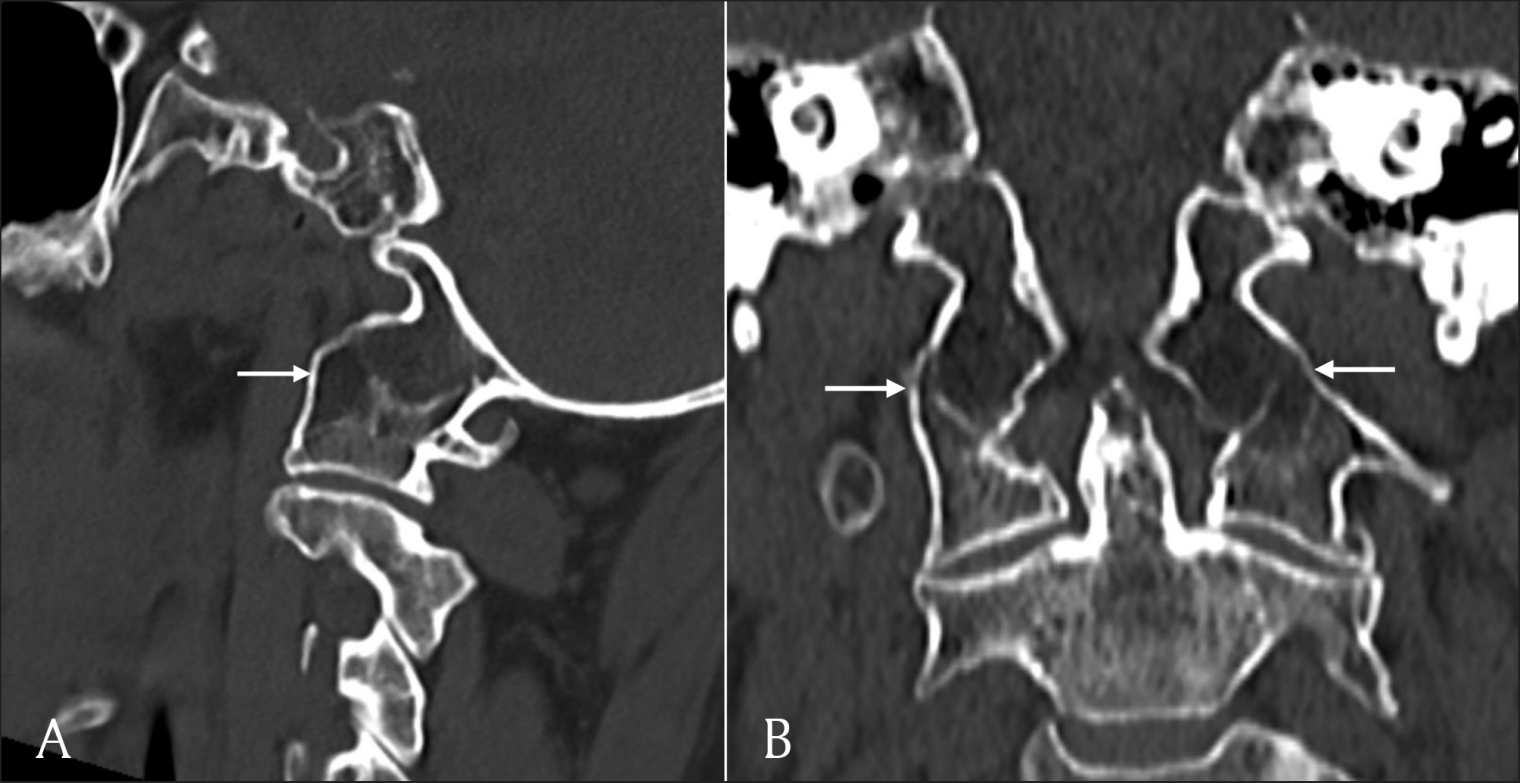
Sagittal **(A)** and coronal **(B)** reconstructions of a CT scan of the cervical spine shows assimilation of the massa lateralis of C1 and the occipital condyle (white arrows).

Pre-operative imaging of the cervical spine also revealed an anatomical variant of the craniocervical junction, atlanto-occipital assimilation of the massa lateralis bilaterally (Figure 4).

The patient was treated with laminectomy of C1 to decompress the spinal canal followed by posterior fixation of C1-C2 for stability.

## Discussion

A retro-odontoid pseudotumor is most commonly associated with rheumatoid arthritis and atlanto-axial subluxation [1]. The pathogenesis of retro-odontoid pseudotumor in rheumatoid arthritis is related to the inflammatory process with pannus formation [2]. In atlanto-axial subluxation, chronic mechanical overuse of the transverse ligament, which provides atlanto-axial stability, results in reactive fibrocartilaginous tissue formation [3]. The pathogenesis is thought to be excessive atlantoaxial movement with increased stress on the transverse ligament, resulting in partial tears and reactive changes to repair the damage [4,5,6]. These reactive changes include hypervascularisation with fibrocartilaginous tissue formation [7]. This process forms a vicious cycle and results in degeneration and abnormal thickening of the transverse ligament with formation of a mass originating from the transverse ligament [1,8].

The transverse ligament provides atlanto-axial stability and often contains fibrocartilaginous tissue as an adaptation to bony compression [9,10]. The hyperintense signal on the T2-weighted images could represent fibrocartilaginous metaplasia or cystic degeneration of the ligament.

In our patient, the degeneration of the transverse ligament could not be attributed to rheumatoid arthritis, as the patient had negative serology. Furthermore, pannus formation as seen with rheumatoid arthritis is accompanied by erosion of bone anterior and/or posterior to the dens. These were absent in this case. Other typical craniocervical pathologies such as meningioma and CPPD had been excluded on post-contrast imaging.

The cause of the excessive tissue formation in this case was attributed to a congenital anomaly, atlanto-occipital assimilation with secondary altered biomechanics in the craniocervical junction. Fusion of the atlas to the occiput in atlanto-occipital assimilation causes the mechanical stress during movement of the head to be transferred to the transverse ligament.

This is in accordance with previous studies that have attributed the development of retro-odontoid pseudotumor, in cases without associated rheumatoid arthritis or atlanto-axial subluxation, to a loss of mobility of the cervical spine, usually adjacent to the level of C1-C2. The altered biomechanics of the cervical spine in these cases resulted in excessive mechanical stress to the atlanto-axial ligaments [1,4,11].

Correct diagnosis of a retro-odontoid pseudotumor due to degeneration of the transverse ligament is important because of its specific treatment, atlantoaxial fixation. The need for dangerous interventions such as biopsy or resection can be avoided. Some cases show spontaneous regression of the fibrocartilaginous tissue after fixation of C1-C2 [12].

MRI is the primary imaging modality to evaluate the extensiveness and possible myelum compression and to demonstrate the ligamentous and fibrocartilaginous characteristics of a retro-odontoid pseudotumor [4]. Computed tomography (CT) lacks specificity to characterize the retro-odontoid mass. However, CT is preferred to detect associated bony anomalies such as fusion of the craniocervical junction and cervical spine. Conventional radiography of the cervical spine with flexion and extension views is useful to evaluate instability in atlantoaxial subluxation.

## Conclusion

Retro-odontoid pseudotumors usually originate from a degenerative transverse ligament due to chronic mechanical stress, most commonly seen in atlanto-axial subluxation. However, previous studies have shown that abnormalities of the cervical spine resulting in decreased mobility, especially adjacent to the level of C1-C2, can also cause excessive mechanical stress on the transverse ligament. In this case report, a congenital anomaly, atlanto-occipital assimilation, resulted in chronic mechanical overuse of the transverse ligament and the development of a retro-odontoid pseudotumor.
